# Synthesis, Spectroscopic and Theoretical Studies of New Quasi-Podands from Bile Acid Derivatives Linked by 1,2,3-Triazole Rings

**DOI:** 10.3390/molecules19022557

**Published:** 2014-02-24

**Authors:** Tomasz Pospieszny, Hanna Koenig, Iwona Kowalczyk, Bogumił Brycki

**Affiliations:** Laboratory of Microbiocide Chemistry, Faculty of Chemistry, Adam Mickiewicz University, Grunwaldzka 6, Poznań 60-780, Poland; E-Mails: koenig@amu.edu.pl (H.K.); iwkow@amu.edu.pl (I.K.)

**Keywords:** bile acids, podands, click chemistry, 1,2,3-triazole ring, PM5 calculations

## Abstract

A novel method for the synthesis of bile acid derivatives has been developed using “click chemistry”. Intermolecular 1,3-dipolar cycloaddition of the propargyl ester of bile acids and azide groups of 1,3,5-tris(azidomethyl)benzene gave a new quasi-podands with 1,2,3-triazole rings. The structures of the products were confirmed by spectral (^1^H-NMR, ^13^C-NMR, and FT-IR) analysis, mass spectrometry and PM5 semiempirical methods. Estimation of the pharmacotherapeutic potential has been accomplished for synthesized compounds on the basis of Prediction of Activity Spectra for Substances (PASS).

## 1. Introduction

Bile acids were isolated from the bile of mammals in 1828 by L. Gmelin. They are produced from cholesterol in the liver and are stored in the gallbladder [1,2,3,4,5,6], from where contraction with feeding releases bile acids into the intestine. The terminal carboxylic acid group at C(17) in the side chain can be conjugated with taurine or glycine. The specific structure of bile acids with a large rigid and curved skeleton, chirality, the orientation of their chemically different polar hydroxy groups (3α; 3α,7α and 3α,7α,12α) toward the center of a concave face, as well as their amphiphilic properties make bile acids and their derivatives very interesting starting materials for the synthesis of macrocyclic molecular dimers, molecular tweezers or cholaphanes [7,8,9,10].

In recent times, much attention was given to the synthesis of molecular pockets, molecular umbrellas and quasi-podands from bile acids [11,12,13,14,15,16,17]. The molecular pockets and umbrellas are composed of two or more facial amphiphiles that are connected to a central, chaining and labile scaffold. On the other hand quasi-podands have a rigid benzyl platform [11,12,13,14,15,16,17]. The potential applications of these compounds include use as delivery vehicles for biological molecules, as molecular containers as well as hydrogelators. Bile acid dimers can be used for the synthesis of macrocyclic compounds as artificial receptors [18,19,20,21,22]. Furthermore some derivatives of bile acids are very good organogelators [23,24,25].

Synthetic or natural podands have acyclic structures where polyether chains are linked to the same binding centre, which can be different heteroatoms, e.g., nitrogen, phosphorus and sulphur. In view of their specific properties, these compounds are so-called open-chain simple analogues of crown ethers and cryptands [26]. Like the above compounds, podand hosts are generally able to form stable complexes with monovalent cations [27]. Synthetic podands have advantages over biological ones, in terms of facile synthesis and molecular structure versatility, therefore the synthesis of compounds containing a steroid skeleton is very interesting and useful. In our previous work, we reported the synthesis and physicochemical properties of new bile acid esters of 1,3,5-tris(bromomethyl)benzene or 1,2,4,5-tetrakis(bromomethyl)benzene and 3α-acetoxy-5β-cholanic acid, 3α,12α-diacetoxy-5β-cholanic acid and 3α,7α,12α-triacetoxy-5β-cholanic acid [17]. To get new quasi-podands we decided to modify the structure of bile acids by introduction of additional 1,2,3-triazole rings using “click chemistry” methods. 

**Scheme 1:**
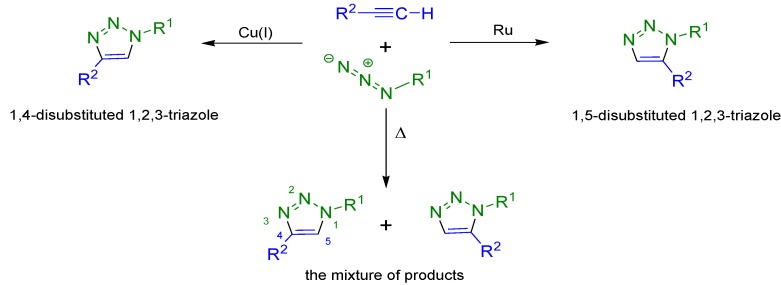
The possible “click” reaction pathways occurring between terminal alkynes and azides.

“Click chemistry” is a relatively new and very attractive trend in modern organic synthesis. It includes a broad spectrum of carbon–heteroatom bond forming reactions that fulfil specified requirements such as high efficiency and selectivity, simple reaction conditions and easy product isolation [28]. Moreover the products of “click chemistry” are stable in various solvents, including water [28,29]. A very important and effective method is the copper(I)-catalyzed 1,3-dipolar cycloaddition (the Huisgen reaction) between azides and terminal alkynes which is regarded as an important example of the “click” reaction. This is a very convenient and simple method of 1,2,3-triazole synthesis. Regioselective cycloaddition reactions lead to the formation of various substituted triazoles. Depending on the catalyst used 1,4-disubstituted triazoles, 1,5-disubstituted triazoles or a mixture of both are formed (Scheme 1) [30,31].

Compounds of this type are very resistant to the hydrolysis, oxidation and reduction conditions of metabolic degradation. Moreover, they can participate in the formation of hydrogen bonds which are crucial interactions in biological systems. In particular, 1,4-disubstitued 1,2,3-triazoles show the ability to participate in hydrogen bonds and dipole interactions [32]. The Cu(I)-catalyzed “click” reaction is thus an extremely useful method to obtain new 1,2,3-triazole derivatives of bile acids [33,34,35,36,37,38,39].

## 2. Results and Discussion

**Scheme 2:**
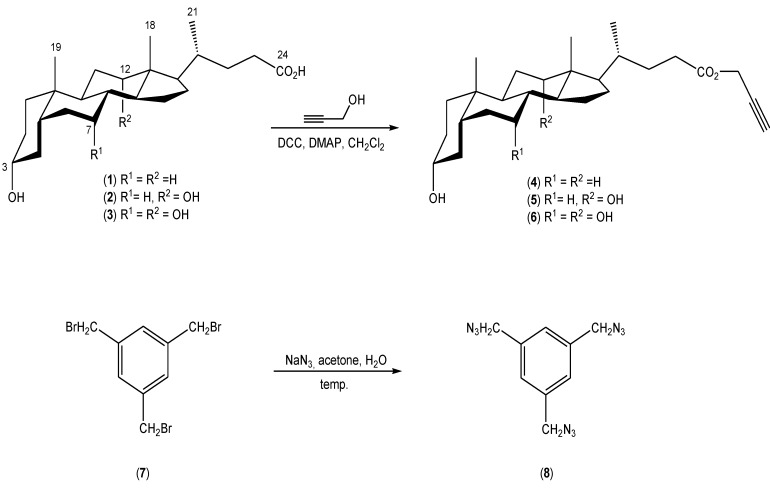
Synthesis of propargyl esters of bile acids **4**–**6** and 1,3,5-tris(azidomethyl)-benzene (**8**).

This work reports the synthesis and physicochemical properties of new quasi-podands linked with 1,2,3-triazole rings from propargyl esters of bile acid derivatives and 1,3,5-tris(azidomethyl)benzene. The propargyl esters of bile acids and 1,3,5-tris(azidomethyl)benzene were prepared according to the literature procedures (Scheme 2) [40,41,42].

**Scheme 3:**
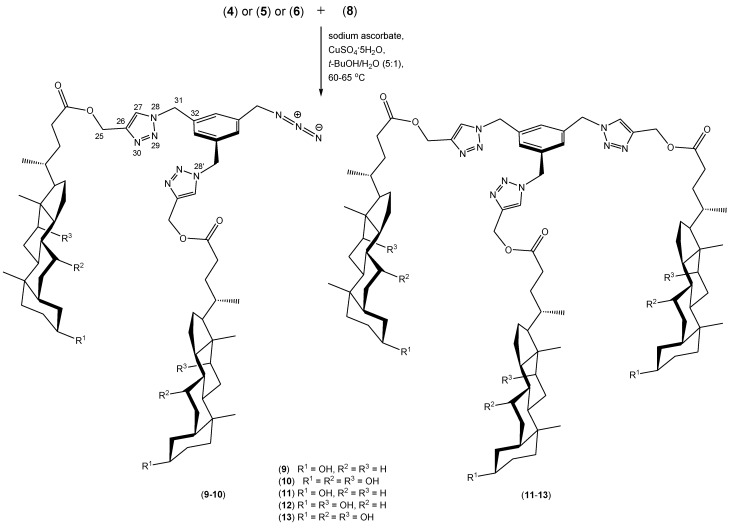
Synthesis of quasi podands of bile acids derivatives **9**–**13** linked by 1,2,3-triazole rings.

The syntheses of compounds **9**–**13** are shown in Scheme 3. We have obtained trisubstituted products **11**–**13** and we have also isolated and characterized two disubstituted products **9**–**10**. These conjugates, in contrast to molecular pockets or umbrellas have a rigid benzyl platform. This allows the easier creation of conformers with an appropriate geometry. The aromatic ring is flat, so it can easily interact with the surface of biopolymers, for example. It performs the function of a specific anchor. Ramírez-López *et al.* have described this type of connection obtained from hormones such as estrone and estradiol and the corresponding azide. These compounds demonstrate differently gelling properties [43,44].

The potential pharmacological activity of synthesized compounds has been determined on the basis of computer-aided drug discovery approaches with the Prediction of Activity Spectra for Substances (PASSs) program. It is based on a robust analysis of structure–activity relationships in a heterogeneous training set currently including about 60,000 biologically active compounds from different chemical series with about 4,500 types of biological activity. Since only the structural formula of the chemical compound is necessary to obtain a PASS prediction, this approach can be used as the earliest stage of an investigation. There are many examples of the successful use of the PASS approach leading to new pharmacological agents [45,46,47,48,49].

Additionally, analyses of the biological prediction activity spectra for the new esters prepared herein are good examples of *in silico* studies of chemical compounds. The biological activity spectra were predicted with PASS for only two synthesized compounds (**9** and **10**). We also selected the types of activity that were predicted for a potential compound with the highest probability (focal activities) (Table 1). According to these data the most frequently predicted types of biological activity are: cholesterol antagonist, hypolipemic and inhibitors of acylcarnitine hydrolase, glyceryl-ether monooxygenase, alkenylglycerophosphocholine hydrolase or alkylacetylglycerophosphatase. We could not determine the potential biological properties of the compounds **11**–**13** because their molecular weight was over 1.200 g/mol.

**Table 1:** Probability ‘to be Active’ (PA) values for the predicted biological activity of compounds **9** and **10**.

Focal Predicted Activity (PA > 0.70)	Compound	
	9	10
Acylcarnitine hydrolase inhibitor	0.79	0.90
Glyceryl-ether monooxygenase inhibitor	0.72	0.84
Alkenylglycerophosphocholine hydrolase inhibitor	0.82	0.82
Biliary tract disorders treatment	0.73	0.81
Hypolipemic	–	0.77
Alkylacetylglycerophosphatase inhibitor	0.76	0.76
Cholesterol antagonist	0.79	0.72

**Figure 1:**
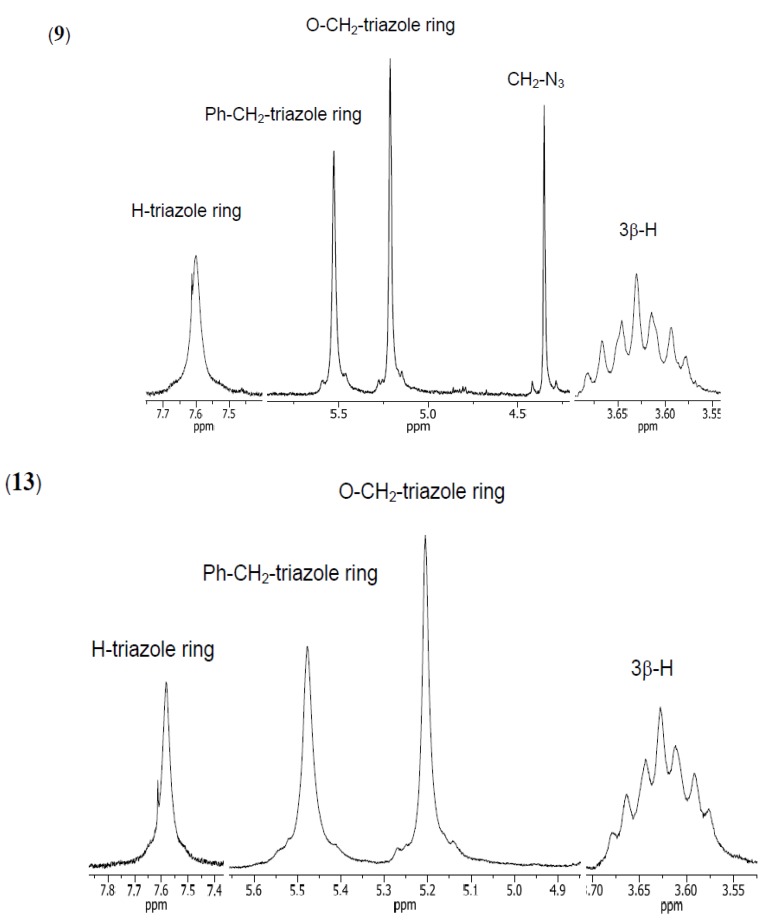
^1^H-NMR spectra in the region (7.8–3.55 ppm) of the most characteristic signals of compounds **9** and **13**.

The structures of all synthesized compounds were determined from their ^1^H- and ^13^C-NMR, FT-IR and ESI-MS spectra. Moreover, PM5 calculations were performed on all compounds [50,51,52]. The ^1^H- NMR spectra of compounds **9**–**10** and **11**–**13** show characteristic multiplets in the 3.68–3.15 ppm range assigned to the C3β–H protons of the steroid skeleton, and two hydrogen singlets ranging from 0.66–0.56 and 0.93–0.81, and characteristic doublets at 0.93–0.88 ppm assigned to CH_3_–18, CH_3_–19, and CH_3_–21, respectively. In the spectra of compounds **10**, **12** and **13** characteristic broad singlets in the 3.96–3.78 ppm range due to the C12β–H protons and singlets in the 3.82–3.61 ppm range for the C7β–H protons (**10** and **13**) were observed. The ^1^H-NMR spectra of **9** and **10** showed a signal at 4.35 ppm for the protons of the –CH_2_–N_3_ group. It is a diagnostic signal, which is not observed in the spectra of **11**–**13**. The signals for two methylene protons of the CO–CH_2_–triazole ring and Ph–CH_2_–triazole ring groups occurred as singlets in the 5.21–5.10 ppm and 5.57–5.48 ppm range, respectively (Figure 1). 

Moreover, the ^1^H-NMR spectra of compounds **9** and **10** showed characteristic, diagnostic singlets at 7.62–7.61 ppm assigned to the two protons of the triazole rings. In the spectra of compounds **11**–**13** were three triazole ring protons in the 8.17–7.58 ppm range were seen. In the ^1^H-NMR spectra of compounds **9**–**13** the most characteristic signals were observed for the aromatic protons of the 1,3,5-trisubstituted benzene. These signals appeared as singlets at 7.21–7.19 and 7.26–7.16 ppm for **9**–**10** and **11**–**13**, respectively. 

The ^13^C-NMR spectra of compounds **9**–**13** show characteristic signals at 12.4–12.0 ppm, 23.4–22.8 ppm, and 18.3–17.5 ppm, which are assigned to CH_3_–18, CH_3_–19, and CH_3_–21, respectively. The carbon atoms of the CO_2_–CH_2_–triazole ring unit resonate in the range of 173.9–173.6 ppm (CO_2_ ) and 65.7–63.1 ppm (CH_2_). In the ^13^C-NMR spectra of compounds **9** and **10** the signals due to CH_2_ in N_3_-CH_2_-Ph group appear in the 53.89–53.88 ppm range. The spectra of compounds **11**–**13** show signals associated with CH_2_ atoms in triazole ring-CH_2_-Ph. The carbon atoms in the triazole ring are located at 143.72–142.20 ppm and 124.94–123.81 ppm, and are assigned to C(26)=C(27)–N(28), respectively.

**Figure 2:**
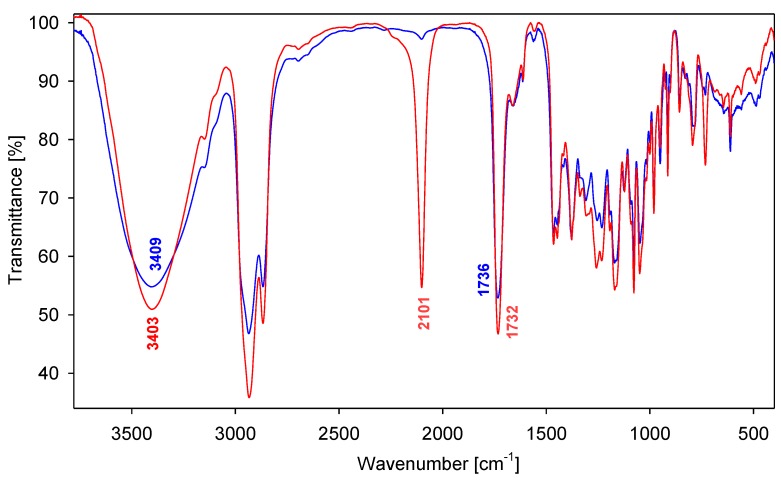
FT-IR spectra of **10** (red) and **13** (blue) in the 3,700–400 cm^−1 ^region.

The most characteristic feature of the FT-IR spectra (film) of all synthesized compounds are bands at 3,441–3,358 cm^−1^ assigned to the *ν*(O-H) stretching vibrations of the O(3)-H, O(7)-H and O(12)-H groups. A very weak hydrogen bond between the OH groups of the steroid skeleton is evidenced by the relatively narrow bands and low intensity. Moreover, two strong characteristic bands in the 1,739–1,732 cm^−1^ and 1,260–1,225 cm^−1^ region are present, which are assigned to the *ν*(C=O) and *ν*(C-O), respectively. For the compounds **9** and **10** are also observed a very strong band at 2,101–2,100 cm^−1^ associated with the presence of *ν*(N=N^+^=N^−^) groups (Figure 2).

**Figure 3:**
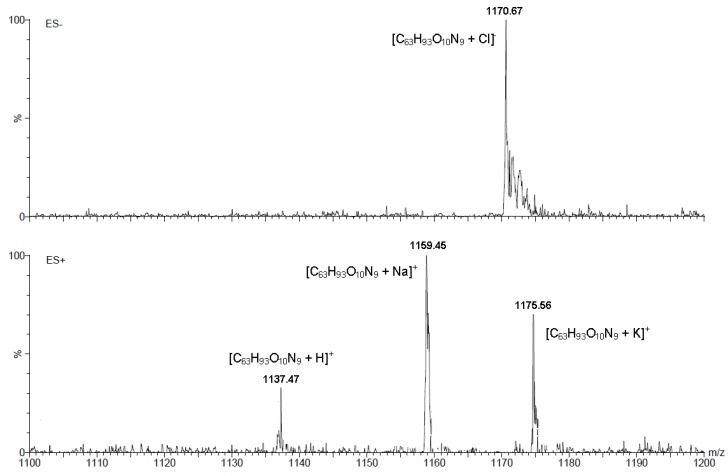
ESI-MS spectrum of conjugate **10**.

The ESI-MS spectra were recorded in methanol. In all cases, the molecular ion [M]^+^ is present, which is associated with the presence in positive ion mode (ES^+^) as well as negative ion mode (ES^−^) of a ion with proton, alkali metals or halides. In Figure 3 we present the ESI-MS spectrum of conjugate **10**. In the spectrum of this conjugate, the ion peaks are observed at *m*/*z* 1,137.47 (35%) [M+H]^+^, *m*/*z* 1,159.45 (70%) [M+Na]^+^ and *m*/*z* 1,175.56 (100%) [M+K]^+^. For this compound in the ESI-MS spectrum in negative ion mode molecular ion is present at *m*/*z* 1,170.67 (100%) [M+Cl]^−^.

PM5 semiempirical calculations were performed using the WinMopac 2003 program. The final heat of formation (HOF), distances of N(28)-N(28') atoms and bond angle of N(28)-C(31)-C(32) of compounds **9**–**13** is presented in Table 2. 

**Table 2:** Heat of formation (HOF) [kcal/mol], distances of N(28)-N(28') atoms [Å] and bond angle [°] of N(28)-C(31)-C(32) of compounds (**9**–**13**).

Compound	Heat of Formation [kcal/mol]	Distance [Å] of N(28)-N(28')	Bond Angle [°] of N(28)-C(31)-C(32)
**9**	−217.30	5.49	110.8
**10**	−382.73	5.49	110.8
**11**	−440.71	5.75	110.8
**12**	−568.23	5.73	111.7
**13**	−694.62	5.46	110.8

**Figure 4:**
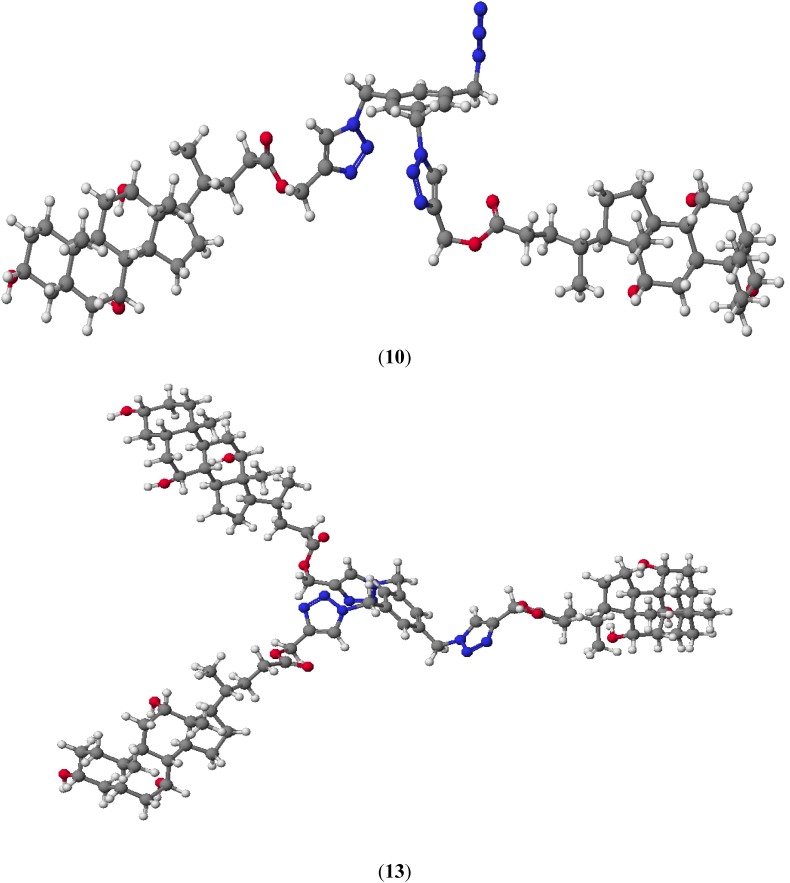
Molecular models of representative compounds **10** and **13** calculated by the PM5 method.

Representative compounds **10** and **13** are shown in Figure 4. The lowest HOF value is observed for cholic acid derivative **13**, where an increasing number of hydroxyl groups facilitate the formation of intramolecular hydrogen bonds. Monosubstituted derivatives of bile acids linked to 1,2,3-triazole rings are not formed because the heat of formation is very high (approx. −66 kcal/mol).

In the all quasi-podands π–π stacking sandwich type interactions between two triazole rings were observed. The calculated interplanar separation is about 5.5 Å. These distances are greater about 1.7 Å in a comparison to the classical π–π stacking interactions because the triazole ring is attached to a rigid aromatic ring which imposes an increasing distance. This is confirmed by the size of the angle between N(28)-C(31)-C(32) atoms (Table 2). Moreover this spatial arrangement of bile acids and 1,2,3-triazole rings can facilitate the formation of stable host-guest complexes.

## 3. Experimental

### 3.1. General

The NMR spectra were measured with a Varian Mercury 300 MHz NMR spectrometer (Oxford, UK), operating at 300.07 and 75.4614 MHz for ^1^H and ^13^C, respectively. Typical conditions for the proton spectra were: pulse width 32°, acquisition time 5 s, FT size 32 K and digital resolution 0.3 Hz per point, and for the carbon spectra pulse width 60°, FT size 60 K and digital resolution 0.6 Hz per point, the number of scans varied from 1200 to 10,000 per spectrum. The ^13^C and ^1^H chemical shifts were measured in CDCl_3 _ or DMSO-*d*_6_ (compound **13**) relative to an internal standard of TMS. Infrared spectra were recorded in the KBr pellets using a FT-IR Bruker IFS 66 spectrometer (Karlsruhe, Germany). The ESI (electron spray ionization) mass spectra were recorded on a Waters/Micromass (Manchester, UK) ZQ mass spectrometer equipped with a Harvard Apparatus (Saint Laurent, QC, Canada), syringe pump. The sample solutions were prepared in methanol at the concentration of approximately 10^−5^ M. The standard ESI-MS mass spectra were recorded at the cone voltage 30 V.

#### Synthesis: General Procedure for the Synthesis of Compounds **9** and **11**

1,3,5-Tris(azidomethyl)benzene (43 mg, 0.177 mmol) was dissolved in mixture of *t*-butanol and water (10 mL, 5:1), propargyl lithocholate (200 mg, 0.482 mmol) was added, the mixture heated at 60–65 °C for 15 min. To the homogenous solution CuSO_4_·5H_2_O (3 mg, 3 mol%) and sodium ascorbate (9 mg, 20 mol%) in water (0.3 mL) were added. The mixture was heated to 60–65 °C for 2 h. The mixture was extracted with chloroform (10 mL), washed with brine (15 mL), and dried over anhydrous MgSO_4_. Evaporation of the solvent and purification of the residue over silica gel (CHCl_3_/EtOAc, 50:1) gave 83.6 mg of product **9** and 133.8 mg of product **11**.

*1-Azidomethylene-3,5-di[4-methylenelitocholate-1-methylene-(1,2,3-triazol-1-yl)]benzene* (**9**): Oil (34%). ^1^H-NMR: δ_H_ 7.61 (s, 2H, triazole ring), 7.20 (s, 3H, Ar–H), 5.53 (s, 4H, Ph–CH_2_–triazole ring), 5.21 (s, 4H, O–CH_2_–triazole ring), 4.35 (s, 2H, CH_2_–N_3_), 3.68–3.58 (m, 2H, 3β–H), 0.91(s, 6H, CH_3_–19), 0.88 (d, *J* = 6.0 Hz, 6H, CH_3_–21), 0.62 (s, 6H, CH_3_–18). ^13^C-NMR (CDCl_3_, TMS, ppm): δ_C_ 174.15, 143.53, 137.94, 136.21, 127.85, 124.08, 71.85, 57.24, 56.47, 55.87, 53.89, 53.71, 42.70, 42.06, 40.40, 40.37, 36.40, 35.81, 35.31, 34.54, 31.04, 30.82, 30.50, 28.19, 27.16, 26.40, 26.21, 24.18, 23.36, 20.80, 18.24, 12.01. FT-IR (film) ν_max_: 3,441, 2,101, 1,735, 1,260, 1,163, 784. ESI-MS (*m/z*): 1,073.46 [C_63_H_93_O_6_N_9_+H]^+^, 1,095.44 [C_63_H_93_O_6_N_9_+Na]^+^, 1,106.67 [C_63_H_93_O_6_N_9_+Cl]^−^.

*1-Azidomethylene-3,5-di[4-methylenecholate-1-methylene-(1,2,3-triazol-1-yl)]benzene* (**10**): Oil (21%). ^1^H-NMR δ_H_ 7.62 (s, 2H, triazole ring), 7.21 and 7.19 (two s, 3H, Ar–H), 5.54 (s, 4H, Ph–CH_2_–triazole ring), 5.19 (s, 4H, O–CH_2_–triazole ring), 4.35 (s, 2H, CH_2_–N_3_), 3.93 (s, 2H, 12β–H), 3.82 (s, 2H, 7β–H), 3.47–3.34 (m, 2H, 3β–H), 0.93 (d, *J* = 6.0 Hz, 6H, CH_3_–21), 0.87 (s, 6H, CH_3_–19), 0.64 (s, 6H, CH_3_–18). ^13^C-NMR (CDCl_3_, TMS, ppm): δ_C_ 174.23, 143.60, 137.77, 136.36, 127.81, 127.32, 123.95, 72.97, 71.84, 68.38, 57.45, 53.88, 53.48, 46.71, 46.37, 41.63, 41.45, 39.49, 39.44, 35.29, 34.72, 34.61, 31.02, 30.75, 30.28, 29.67, 28.13, 27.55, 26.35, 23.20, 22.45, 17.24, 12.40, 0.99. FT-IR (film) ν_max_: 3,403, 2,100, 1,732, 1,258, 1,169, 732. ESI-MS (*m/z*): 1,137.46 [C_63_H_93_O_10_N_9_+H]^+^, 1,159.46 [C_63_H_93_O_10_N_9_+Na]^+^, 1,175.59 [C_63_H_93_O_10_N_9_+K]^+^, 1,170.68 [C_63_H_93_O_10_N_9_+Cl]^−^.

*1,3,5-Tris[4-methylenelitocholate-1-methylene-(1,2,3-triazol-1-yl)]benzene* (**11**): Oil (54%). ^1^H-NMR: δ_H_ 7.58 (two s, 3H, triazole ring), 7.20 and 7.16 (s, 3H, Ar–H), 5.48 (s, 6H, Ph–CH_2_–triazole ring), 5.21 (s, 6H, O–CH_2_–triazole ring), 3.66–3.59 (m, 3H, 3β–H), 0.91(s, 9H, CH_3_–19), 0.88 (d, *J* = 6.1 Hz, 9H, CH_3_–21), 0.62 (s, 9H, CH_3_–18).^13^C-NMR (CDCl_3_, TMS, ppm): δ_C_ 174.13, 143.71, 136.76, 127.68, 123.91, 71.82, 57.35, 56.48, 55.89, 53.34, 42.71, 42.06, 40.15, 40.40, 36.42, 35.81, 35.31, 35.30, 34.55, 31.05, 30.84, 30.52, 28.19, 27.16, 26.40, 24.18, 23.36, 20.81, 18.25, 12.01. FT-IR (film) ν_max_: 3,358, 1,732, 1,225, 1,163, 757. ESI-MS (*m/z*): 1,487.05 [C_90_H_135_O_9_N_9_+H]^+^, 1,509.04 [C_90_H_135_O_9_N_9_+Na]^+^, 1,525.08 [C_90_H_135_O_9_N_9_+K]^+^, 1,521.01 [C_90_H_135_O_9_N_9_+Cl]^−^.

*1,3,5-Tris[4-methylenedeoxycholate-1-methylene-(1,2,3-triazol-1-yl)]benzene* (**12**): Oil (43%). ^1^H-NMR: δ_H_ 8.02 (s, 3H, triazole ring), 7.17 (s, 3H, Ar–H), 5.49 (s, 6H, Ph–CH_2_–triazole ring), 5.19 (s, 6H, O–CH_2_–triazole ring), 3.96 (s, 3H,12β–H), 3.61-3.59 (m, 3H, 3β–H), 0.92 (d, *J* = 6.1 Hz, 9H, CH_3_–21), 0.91 (s, 9H, CH_3_–19), 0.66 (s, 9H, CH_3_–18). ^ 13^C-NMR (CDCl_3_, TMS, ppm): δ_C _174.08, 143.72, 136.77, 127.73, 123.81, 73.05, 71.70, 57.42, 53.32, 48.24, 47.10, 46.46, 42.04, 35.97, 35.23, 35.10, 34.08, 33.58, 31.42, 31.07, 30.76, 30.45, 28.64, 27.50, 27.09, 26.12, 23.64, 23.11, 17.24, 12.67. FT-IR (film) ν_max_: 3,434, 1,739, 1,255, 1,167, 758. ESI-MS (*m/z*): 1,535.04 [C_90_H_135_O_12_N_9_+H]^+^, 1,557.02 [C_90_H_135_O_12_N_9_+Na]^+^, 1,568.98 [C_90_H_135_O_12_N_9_+Cl]^−^, 1,612.96 [C_90_H_135_O_12_N_9_+Br]^−^.

*1,3,5-Tris[4-methylenecholate-1-methylene-(1,2,3-triazol-1-yl)]benzene* (**13**): oil (55%). ^1^H-NMR: δ_H_ 8.17 (s, 3H, triazole ring), 7.26 (s, 3H, Ar–H), 5.57 (s, 6H, Ph–CH_2_–triazole ring), 5.10 (s, 6H, O–CH_2_–triazole ring), 4.33 (d, 3H, *J* = 3 Hz, 7α–OH), 4.11 (d, 3H, *J* = 3 Hz, 3α–OH), 4.01 (d, 3H, *J* = 3 Hz, 12α–OH), 3.78 (s, 3H, 12β–H), 3.61 (s, 3H, 7β–H), 3.21–3.15 (s, 3H, 3β–H), 0.90 (d, 9H, *J* = 6.1 Hz, CH_3_–21), 0.81(s, 9H, CH_3_–19), 0.56 (s, 9H, CH_3_–18). ^13^C-NMR (DMSO_d6_, TMS, ppm): δ_C_ 173.11, 142.20, 137.12, 127.54, 124.94, 72.86, 70.99, 70.44, 66.25, 56.95, 52.30, 46.09, 45.76, 41.52, 41.37, 35.32, 35.02, 34.88, 34.40, 30.65, 30.58, 30.41, 28.52, 28.51, 27.28, 26.21, 22.82, 22.64, 16.88, 12.29. FT-IR (film) ν_max_: 3,409, 1,736, 1,255, 1,162, 759. ESI-MS (*m/z*): 1,584.07 [C_90_H_135_O_15_N_9_+H]^+^, 1,606.03 [C_90_H_135_O_15_N_9_+Na]^+^, 1,620.96 [C_90_H_135_O_15_N_9_+K]^+^, 1,616.98 [C_90_H_135_O_15_N_9_+Cl]^−^, 1,660.95 [C_90_H_135_O_15_N_9_+Br]^−^.

## 4. Conclusions

In conclusion, five new bile acid esters **9**–**13** were prepared from propargyl esters of lithocholic, deoxycholic and cholic acid and 1,3,5-tris(azidomethyl)benzene *t*-butanol/water mixture in the presence of sodium ascorbate and CuSO_4_**·**5H_2_O at 65 °C. These new compounds were characterized by spectroscopic and molecular structure methods. These bile acid esters may find applications in molecular recognition, supramolecular chemistry, and in pharmacology.
